# Supplementary material for: In vitro organ culture protocol for intact urogenital systems supporting gonadal differentiation
Source: PLoS One. 2025 Jul 29;20(7):e0327930. doi: 10.1371/journal.pone.0327930 (PMC12306756; doi:10.1371/journal.pone.0327930)
Supplement: S1 File — (PDF) [file pone.0327930.s001.pdf]

In vitro organ culture of intact urogenital systems supporting gonadal differentiation

Sarah Whiteley[1], Clare Holleley[2], Arthur Georges[1]

[1]University of Canberra; [2]CSIRO

Sarah Whiteley
University of Canberra

Protocol Info: Sarah Whiteley, Clare Holleley, Arthur Georges . In vitro organ culture of intact urogenital systems supporting gonadal differentiation. **Protocol exchange** **https://protocols.io/view/in-vitro-organ-culture-of-intact-urogenital-system-dt5g6q3w**

Created: December 08, 2024

Last Modified: December 08, 2024

Protocol Integer ID: 114568

Keywords: organ culture, sex determination, sex differentiation

Funders Acknowledgement:
Australian Research Council
Grant ID: DP170101147
Australian Research Council
Grant ID: DP220101429
CSIRO
Grant ID: Research Plus Postgraduate Award

Abstract

This protocol is for the *in vitro* culture of intact embryonic urogenital systems using an agarose substrate. This protocol has been optimised for the model reptile species, *Pogona vitticeps*, but could be adapted for other species. This protocol supports gonad differentiation from bipotential to ovaries or testes that are distinguishable morphologically and by gene expression patterns. Gross morphology is well maintained even during the longest culture duration (19 days) such that the gonads can be dissect from the surrounding mesonephros, improving utility for downstream sequencing applications.

Materials

- Leibovitz's
  L-15 medium with L-glutamine, without phenol red (Thermofisher, Cat: 21083207)
- 100 x Anti-Anti (Gibco, Cat: 15240062)
- Fetal bovine serum (Gibco, Cat: A3382001), or purchase already charcoal stripped if available
- Agarose (Merck, Cat: A9539)
- Centre well culture dishes (Corning Costar, Cat: CLS3260)
- Positive displacement pipette (Rainin, Cat: MR-1000)
- Capillary-pistons (Rainin, Cat: 17008609)
- Dextran coated charcoal (Sigma, Cat: C6241-5G)
- Sterile PBS or ultrapure water
- Transfer pipettes (Livingstone, Cat: PTP03)

Note

Phenol red is a weak estrogen mimic and can influence gonad differentiation (Shoemaker-Daly *et al.*, 2010) so must be avoided in all reagents.

Fetal bovine serum contains hormones so must be charcoal stripped if it is not available to purchase already stripped. Standard serum can be stripped using dextran coated charcoal according to the manufacturer's protocol.

Equipment

- Dissection microscope
- Biological safety cabinet
- Temperature controlled cell culture incubators with 5% C02
- Curved plastic moulds
- Forceps (standard for manipulating the plastic moulds)
- Forceps (ultrafine tips for embryo dissections, such as Dumont style 5, Cat: T05-922)
- Scissors
- Petri dishes (for dissections, required size depends on size of species used)

Before start

This protocol has been developed and optimised using *Pogona vitticeps* as a model system. Before starting, species specific characteristics should be carefully considered. The timing of explantation must be carefully balanced with the duration of the culture period. The culture must be maintained long enough for gonad differentiation to occur, but not so long as to potentially compromise the tissue. This relies on knowing the timing of gonad development and differentiation in the target species.
As the agar moulds are made in-house, their size can be modified as required to accommodate urogenital systems. For *P. vitticeps*, the moulds are approximately 5x3x3mm. The size required relates to the size of the urogenital system at time of explantation as the overall size of the organ does not noticeably change during the culture period.

Culture Medium

1 To prepare complete culture medium, add 1 mL of charcoal stripped fetal bovine serum and 1 mL of Anti-Anti to 10 mL of L-glutamine medium with no phenol red.

> **Note**
>
> For ease of use, L-15 medium can be stored in 10mL aliquots, and 1mL aliquots each of charcoal stripped FBS and Anti-Anti added to make 12mL total volume of complete media.

Agar Moulds

2 Dissolve 187 mg of agar in 12 mL of L-15 medium (no added Anti-Anti or FBS) in a microwave on low power (the agarose has a low melting point).

3 Using a positive displacement pipette, distribute 500 µL in the centre well of the culture dish making sure the whole surface is covered and there are no bubbles.

4 Before the agar solidifies, use forceps to place the plastic mould in the centre of the dish to form the well for the urogenital system.

> **Note**
>
> Because the agar has a low melting point, the stock will readily re-solidify when preparing the dishes. To keep the agar stock warm while preparing the dishes, heat water in a bottle to float the 50 mL falcon tube in, and wrap with foil to help retain warmth. If the stock solidifies, it can be re-melted.

Dissection of whole urogenital systems

5

> **Note**
>
> For *P. vitticeps*, whole urogenital systems are explanted at stage 6 (bipotential gonad) and cultured until they would have reached stage 12 (differentiated gonad). At 36°C, embryos are sampled 9 days post lay, and are cultured for 9 days (or 18 days from lay). At 28°C, embryos are sampled 16 days post lay, and are cultured for 19 days (or 35 days from lay). The timing of explantation, the culture duration, and incubation temperatures, will depend on the species characteristics.

6 Pre-prepare appropriate number of culture dishes and fill with 500 µL of pre-warmed complete culture media.

Note

This volume is appropriate for high temperature incubations at 36°C where the media can quickly evaporate between changes. This volume may need to be adjusted depending on the temperatures used.

7 Clean the dissection area, and all equipment (scissors to cut the egg, and two pairs of forceps for the dissection).

8 Remove the embryo from the egg and humanely euthanise prior to commencing the dissection.

Note

P. vitticeps has a leathery eggshell that can be cut with scissors. Removal of the embryo will need to be adapted for species with different eggshell characteristics, or for viviparous species. To keep the dissection dish clean, initially remove the embryo from the egg in a petri dish, then transfer the embryo only into a clean dish.

9 Transfer the embryo to a clean dish, and submerge in sterile PBS or ultrapure water to limit risk of contamination in the cultures.

Note

Use single use transfer pipettes for each embryo and keep the lid on the PBS/water.

10 Take any other tissue or blood samples that may be required.

11 Remove the intact urogenital system from the embryo (gonad-kidney complex) and transfer to agar dish.

Note

The transfer should be made as quickly as possible, and the culture dish lid should only be removed to place the organ to minimise any potential for contamination.

12 Check the organ is orientated in the agar dish the same way it was positioned in the embryo.

Note

The organs may need to be gently pushed into the well as they sometimes float in the culture media.

13 Transfer organ cultures to C02 incubator set to an appropriate temperature.

Note

Any movement of the cultures should be made as carefully as possible as the organs can float and potentially move out of the well. Where possible, keep transfer distances as short as possible between the microscope, the incubators, and the biological safety cabinet.

Culture Maintenance

14

Note

Organ cultures should be allowed to establish prior to commencing any experimental treatments.
Media should be refreshed every second day, so start treatments from the second day after the cultures have been set up. For example, if the cultures were explanted on Monday, they should be left untouched until Wednesday, when the media can be changed, and experimental treatments can be applied.

15 Prepare complete culture media and pre-warm

16 Use a sterile pipette tip to remove the medium from the dish

17 Replace with 500 µL of medium, including any experimental treatments as required

Note

Always pipette slowly to avoid disrupting the organ. Pipette culture media in and out of the dishes from the side. Never directly touch the organ.

Culture Arrest

18 The duration of the culture period should be pre-determined depending on the species-specific characteristics. The cultures should be arrested so that the timing is synchronised with when the culture was next due to be changed. This means that if any treatments were applied to the culture exposure has been maximized.
The overall morphology of the organs can be variable at the end of the culture. This depends on the quality of the initial dissection, and effects any treatments applied may have had. The gonads should be distinguishable from the kidney under a microscope, and so can be carefully dissected for use in downstream applications as appropriate.
For RNA-seq or qPCR, gonads can be dissected and snap frozen.
For histology, or other IHC techniques, the whole urogenital system can be preserved (typically in 4% PFA-PBS at 4°C). It is recommended to leave the system intact for any morphological

techniques as this allows the tissues to be handled by the kidney, keeping the gonadal tissues protected.

Additional notes

19

- The initial dissection is very important for the success of the culture. As much as possible, minimise any tugging on the UGS to remove it from the body cavity. For *P. vitticeps*, the posterior region of the kidney becomes long and narrow, extending well into the base of the tail. For the purposes of the organ culture, the posterior region is not important, so can be cut from where it begins to narrow. This also keeps non-target tissue to a minimum in the culture system.
- A microscope set up with foot pedal focus controls will greatly facilitate the dissections
- The embryos are dissected outside of a biological safety cabinet, however the cultures do not appear susceptible to contamination despite this.
- The dissection is easier if the embryo is on a dark background. Custom dissection dishes can be made using melted paraffin wax dyed black with a wax dye, and set into a petri dish. These can be used for multiple dissections if cleaned in between samples.
- Avoid grasping the tissue with forceps where possible. Transferring the organ system to the agar dish is best done using a “forklift” type technique, where the forceps are carefully slid underneath the organ and it is lifted up.
- Positioning the urogenital system in the agar dish on top of a black surface makes it easier to see that the tissue is oriented correctly.
- The culture medium should always be pre-warmed. Never apply cold culture medium to the organ cultures.
- Experimental treatments on *P. vitticeps* organ cultures have applied drugs to the cultures at every medium change (after the initial establishment period) for the duration of the culture period (depending on incubation temperature). All drugs trialled appear to be well tolerated by the cultures.

Protocol references

Martineau, J. *et al.* (1997) Male-specific cell migration into the developing gonad, *Current Biology*, 7(12), pp. 958–968. doi:10.1016/S0960-9822(06)00415-5.

Mork, L. and Capel, B. (2013) Conserved action of β-catenin during female fate determination in the red-eared slider turtle, *Evolution and Development*, 15(2), pp. 96–106. doi:10.1111/ede.12020.

Mork, L., Czerwinski, M. and Capel, B. (2014) Predetermination of sexual fate in a turtle with temperature-dependent sex determination, *Developmental Biology*, 386(1), pp. 264–271. doi:https://doi.org/10.1016/j.ydbio.2013.11.026.

Shoemaker-Daly, C.M. *et al.* (2010) Genetic network underlying temperature-dependent sex determination is endogenously regulated by temperature in isolated cultured *Trachemys scripta* gonads, *Developmental Dynamics*, 239(4), pp. 1061–1075. doi:10.1002/dvdy.22266.

Acknowledgements

The testing and now frequent use of this organ culture method came about because of the COVID-19 pandemic restricting the availability of the Biopore membranes. We are grateful for this unintended, yet ultimately positive outcome. We thank Chelsea Steele and her animal husbandry team at the University of Canberra for their care of the *P. vitticeps* animal colony that supports our research. We acknowledge the
contributions of Blanche Capel and her team in being the first to adopt this agar slab method in reptiles, and for providing the methodological foundation which we based this protocol on. We also thank members of Team Pogona, past and present, for their support throughout the development of this protocol.
