# Supplementary material for: In vitro organ culture protocol for intact urogenital systems supporting gonadal differentiation
Source: PLoS One. 2025 Jul 29;20(7):e0327930. doi: 10.1371/journal.pone.0327930 (PMC12306756; doi:10.1371/journal.pone.0327930)
Supplement: Attachment — Submitted filename: Response to Reviewers.docx [file pone.0327930.s007.docx]

**Manuscript PONE-D-24-57396: *In vitro* organ culture protocol for intact urogenital systems supporting gonadal differentiation**

**Response to reviewers**

We thank the Editor and the two Reviewers for taking the time to consider our manuscript for publication in PLOS One. The manuscript was well received by Reviewer 1 who didn’t provide any suggestions for the manuscript, stating that the protocol provides “a reliable method for studying gonadal differentiation in non-model species.”

We have responded in detail to the comments from Reviewer 2 in BLUE, and actions we have taken to address these comments are in RED. In the amended manuscript the amendments have been made using tracked changes as requested by the editorial team, along with a clean revised version. All referenced line numbers correspond to the tracked changes version of the manuscript.

**Reviewer 2 Response**

We appreciate the thoroughness of the response from Reviewer 2. We note that the majority of the comments share a theme in that there are limitations of our present method that are inherent to *ex ovo* culture systems. It is not possible to ever perfectly replicate the *in vivo* environment in culture.

We acknowledged this in the original submission on lines 187-189, where we stated that “we expect the in vitro cultures will not completely recapitulate the expression patterns of gonads developing *in situ*, as no culture system can ever capture the biological complexities present in an intact and normally developing embryo.”

This includes the absence of any signals that may originate from the hypothalamus (comment 1) and other endocrine signalling factors (comment 3) that may mean that there are “subtle changes” in the organ culture (comment 2).

ACTION: In order to address the overarching concerns central to these comments, we have added the following lines to the revised manuscript:

“While no in vitro culture system can ever completely recapitulate the environment experienced *in vivo*, the goal is to develop techniques able to sufficiently capture the biological complexity and overcome obstacles present in the in vivo system.” (lines 57-60).

“Although there are limitations inherent to *in vitro* culture systems, in such instances they present the only viable alternative for experimentation.” (lines 71-72).

The other concern raised by Reviewer 2 consistent across three comments was that additional validation may shed more light onto the efficacy of the culture method. This includes hormonal assays (comment 5), single cell RNA-sequencing (comment 6), and matrix-based hydrogels (comment 8).

We agree that there are many options available for validating and optimising *in vitro* culture protocols, as described in lines 241-249 of the original submission. We suggest the utility of whole transcriptome sequencing but also acknowledge other more cost-effective approaches like qPCR, and other morphology based techniques alongside histology, such as immunohistochemistry.

One of the major advantages of our method is its simplicity and low cost (mentioned in lines 35-36), making it accessible to all researchers compared to techniques that rely on expensive consumables like biopore membranes and hydrogel matrixes.

ACTION: To highlight the current limitations of our presented method, we have added the following lines and suggest additional avenues for optimisation of the technique, we have added the following lines:

“Many other sequencing techniques are available for validating expression profiles, for example single cell sequencing, but are often cost prohibitive.” (lines 248-249).

“Other assays assessing hormonal function could also be considered depending on research requirements.” (lines 254-255).

**Other comments from Reviewer 2**

1. In vivo, gonadal development is influenced by systemic factors such as hormones from the hypothalamus-pituitary-gonadal (HPG) axis. This in vitro system, while valuable, lacks these interactions, which may lead to incomplete or altered differentiation patterns. It's important to acknowledge these limitations and consider external supplementation with hormones or signaling molecules to mimic in vivo conditions fully.

We thank Reviewer 2 for their comment and agree that any *in vitro* system has inherent limitations and will never be able to fully recapitulate the *in vivo* environment, as addressed in response to comments above.

We also note that the organ culture is not fully separated from the entire HPA (hypothalamus-pituitary-adrenal) axis because the intact UGS is explanted such that the adrenal is still present. This is an advantage to previous methods where the gonad was cultured in isolation, so was completely removed from the whole axis. We also note that there is limited evidence for the role of the HPA axis in reptile sex determination (reviewed in Castelli *et al*, Biological Reviews, 2020).

Any signalling molecules potentially originating from the HPA axis are poorly characterised in reptiles, so it would be difficult to determine any necessary supplementation to the culture medium. Additionally, as outlined in this paper, the gonads of multiple reptile species can differentiate *in vitro* suggesting that any influence from the HPA axis is not critical to this process.

ACTION: To address this suggestion from Reviewer 2, we have added the following statement to lines 90-91: “It also removes the gonads from the hypothalamic-pituitary-adrenal (HPA) axis, which may influence gonad growth and development”.

We also added the following on lines 102-103: “While culture of the whole HPA axis is impossible, the culture of the intact urogenital system at least includes the adrenal, which may enhance gonad growth and development in culture.”

4. The study highlights that Biopore membranes fail to maintain gonad morphology, but the agar slab method does not fully replicate in vivo development. The extent to which these morphological differences affect downstream analyses (e.g., gene expression) is not deeply explored. The expression of sex-specific genes in cultured gonads does not fully match that observed in in situ development, particularly in 36°C-induced sex-reversed females. The study suggests that high temperatures may mask sex-specific gene expression, but alternative explanations should be considered, such as differences in developmental timing or stress responses in vitro. These factors could potentially influence gene expression and should be further investigated.

Throughout the manuscript we have been careful to never claim that our agar slab method fully replicates the *in vivo* environment. Indeed, we have drawn attention to the fact this is not possible in several parts of the manuscript, and have made further additions to the revised paper in response to other comments from Reviewer 2 in relation to this.

We do agree with Reviewer 2 that temperature complicates the gene expression patterns observed in the 36°C sex reversed females, as stated in the original submission: “This may be because high temperatures mask some of the sex-specific gene expression patterns, and there appears to be differences in the timing of gene expression compared to gonads developing in situ.” (lines 214-216).

On lines 241-243 we also stated that: “Even for in situ gonads, expression patterns can be complicated, and with the addition of the temperature influences, and be complicated even further. Because of this, care should be taken if using gene expression profiling in general, but particularly for in vitro organ cultures.”

ACTION: To further highlight the complicating effects of temperature, we have added the following: “This is especially important for species with thermosensitive sex determination systems where the influence of temperature on sex differentiation is fundamentally linked.” (lines 244-246).

7. The study does not clearly state the number of biological replicates used for gene expression analysis. Given the complexity of temperature-induced sex reversal, a larger sample size would help determine whether observed trends are biologically meaningful or due to individual variability.

ACTION: We have included sample sizes for the different groups on lines 172, 189, 262, 266. The sample sizes are comparable to previous studies, particularly for transcriptomics (see Whiteley *et al.,* 2021, 2022 for examples).

9. The study optimizes culture conditions based on previous P. vitticeps staging but does not test whether earlier or later developmental stages might yield better results. A more detailed assessment of optimal culture windows could improve the model.

As stated on lines 133-135 of the original submission, we did test the influence of explanting tissue earlier in development (stage 4). We noted on lines 16-169 that we chose stage 6 to “keep the culture duration to a minimum”. Similarly, we did not explant later in development as previous work on the species showed that differentiation can occur as early as stage 8, and we did not want to risk explantation when the gonad was committed to a sexual fate (Whiteley *et al*., Scientific Reports, 2018).
